# Intradural metastasis to the cauda equina found as the initial presentation of breast cancer: a case report

**DOI:** 10.1186/s13256-019-2155-z

**Published:** 2019-07-20

**Authors:** Keita Koyama, Hiroshi Takahashi, Masahiro Inoue, Akihiko Okawa, Arata Nakajima, Masato Sonobe, Yorikazu Akatsu, Junya Saito, Shinji Taniguchi, Manabu Yamada, Keiichiro Yamamoto, Yasuchika Aoki, Takeo Furuya, Masao Koda, Masashi Yamazaki, Seiji Ohtori, Koichi Nakagawa

**Affiliations:** 1 Department of Orthopaedic Surgery, Toho University Sakura Medical Center, 564-1, Shimoshizu, Sakura City, Chiba 285-8741 Japan; 2 Department of Orthopaedic Surgery, Chiba University Graduate School of Medicine, 1-8-1, Inohana, Chuoku, Chiba City, Chiba 260-8677 Japan; 3 Department of Orthopaedic Surgery, National Hospital Organization Chiba Medical Center, 4-1-2, Tsubakimori, Chuoku, Chiba City, Chiba 260-8606 Japan; 4 Department of Orthopaedic Surgery, Chiba Eastern Medical Center, 3-6-2, Okayamadai, Togane City, Chiba 283-8686 Japan; 5 Department of Orthopaedic Surgery, University of Tsukuba, 1-1-1, Tennodai, Tsukuba City, Ibaragi 305-8575 Japan

**Keywords:** Cauda equina tumor, Intradural metastasis, Breast cancer, Case report

## Abstract

**Background:**

Intradural extramedullary spinal metastasis is a relatively rare condition. Furthermore, there are few reports with the initial presentation being a neurological symptom from an intradural metastasis. We report a case of a patient with metastasis to the cauda equina from breast cancer found due to neurological symptoms as the initial presentation.

**Case presentation:**

A 76-year-old Japanese woman who was previously healthy presented to our hospital with bilateral severe buttock and lower extremity pain without any history of injury. A solitary intradural cauda equina mass was found by magnetic resonance imaging at the L2/3 level, and we suspected a schwannoma initially. The patient hoped to undergo surgery due to the severe pain. However, the chest computed tomographic scan obtained to assess the patient’s general status showed the suspected breast cancer of the left side and a lung metastasis. Hence, we considered the possibility of cauda equina tumor metastatic from the breast cancer. We performed an L1–3 laminectomy and tumor extirpation. The pathology revealed adenocarcinoma. After surgery, she had relief from pain, and her status remained satisfactory until she died 9 months after surgery.

**Conclusions:**

It is difficult to clarify whether the cauda equina tumor is benign or malignant based only on Magnetic resonance imaging findings. Clinicians should consider the possibility of metastasis when planning the surgery for intradural cauda equina tumor extirpation.

## Background

Metastasis to the spinal vertebral column is relatively common. Sometimes patients have severe pain or a neurological deficit through compression of the spinal cord or the cauda equina because of structural instability or pathological fracture. By contrast, the major pathology of intradural tumor in the cauda equina is schwannoma, and most are benign [1, 2]. Otherwise, intradural extramedullary metastasis is an extremely rare condition [3–5]. There are few reports of metastasis to the cauda equina, and the origins were kidney, lung, and breast cancer [6–11]. However, it is infrequent that a cancer initially presents with neurological symptoms due to compression of the cauda equina. We report a case of a patient with metastasis from breast cancer to the cauda equina initially presenting with neurological symptoms.

## Case presentation

**Fig. 1:**
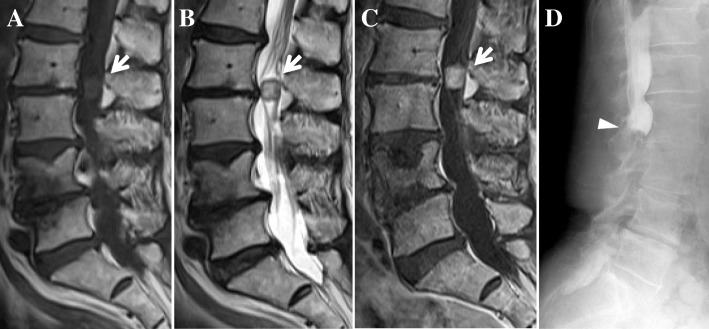
**a–c** Sagittal magnetic resonance imaging. **a** T1-weighted image. **b** T2-weighted image. **c** T1-weighted gadolinium-enhanced image. Intradural extramedullary mass at the level of L2–L3 showing T1-low, T2-high signals enhanced uniformly with gadolinium (*arrow*). **d** Lateral myelography showing a total block of contrast below the level of L2–L3 and capping of contrast by the mass (*arrowhead*)

**Fig. 2:**
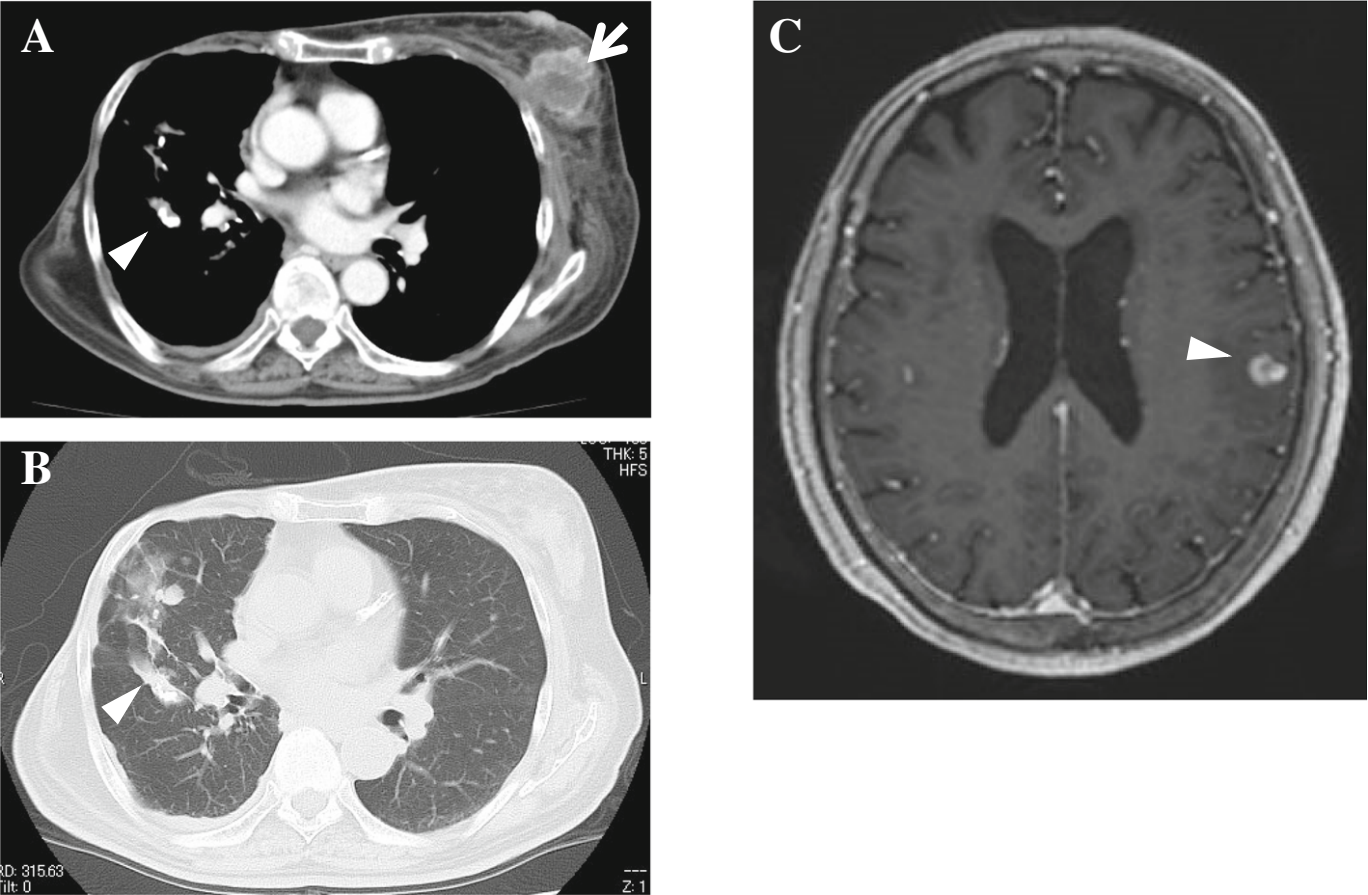
**a** Contrast-enhanced computed tomography (CT) mediastinal window showing the suspected breast cancer on the left side (*arrow*) and lung metastasis (*arrowhead*). **b** Plain CT lung window showing the suspected lung metastasis (*arrowhead*). **c** Axial magnetic resonance imaging scan of the brain with T1-weighted gadolinium contrast enhancement showing one small coin lesion in the temporal lobe of the left side with a diameter of about 5 mm, a suspected metastasis (*arrowhead*)

**Fig. 3:**
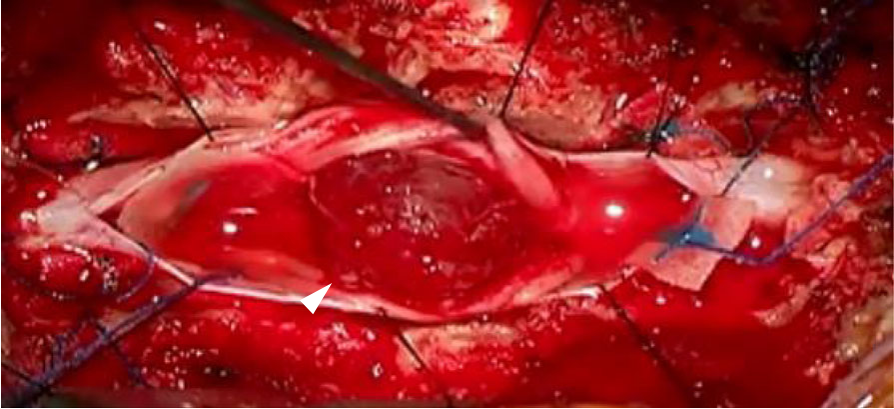
Intraoperative finding. *Arrowhead* indicates the tumor

After 1 month of conservative treatment elsewhere, a 76-year-old Japanese woman who was previously healthy presented at our hospital with bilateral severe buttock and lower extremity pain, without a history of injury. She had a history of diabetes, hypothyroidism, and right breast cancer treated surgically 30 years previously. She had no appreciable familial or psychosocial history. Examination revealed the following: severe pain in the buttocks and posterior femoral area, positive straight-leg-raising tests at 30 degrees bilaterally, positive Valleix pain point and superior gluteal nerve pain point tests bilaterally, and negative femoral nerve-stretching tests bilaterally. The patient’s visual analogue scale (VAS; 100 mm) scores for lower extremity pain and numbness were 100/100 mm. By contrast, she had no motor deficit or dysfunction of the bladder or bowel. X-ray findings showed mild spondylosis. Magnetic resonance imaging (MRI) revealed a solitary intradural extramedullary mass at L2/3 with low T1, high T2, and uniform contrast enhancement with gadolinium (Fig. 1a–c). Myelography showed a total block of contrast below L2/3 and capping of contrast by the mass (Fig. 1d). The diagnosis was a solitary intradural extramedullary cauda equina tumor (a suspected schwannoma). The patient desired tumor extirpation because of the severe pain, so we evaluated her general status. A chest computed tomographic scan showed a suspected left breast cancer and lung metastasis (Fig. 2a, b). Brain MRI showed one small mass in the temporal lobe of the left side with a diameter of about 5 mm, a suspected metastasis (Fig. 2c). We considered a cauda equina tumor metastatic from the breast cancer. After obtaining informed consent, we performed an L1–3 laminectomy and tumor extirpation. Bloody cerebrospinal fluid was observed after the dura mater incision was made. The tumor was involved with the intact cauda equina, and careful division of adhesions was performed. After cutting the filum terminale (conglutinated with the tumor), the tumor was extirpated *en bloc* (Fig. 3).

Postoperatively, the patient experienced pain relief. The pathology of the metastasis was adenocarcinoma. The result of cerebrospinal fluid cytology was negative. The patient started chemotherapy for breast cancer. At the 3-month postoperative follow-up, her VAS score was decreased for the lower extremity pain (from 100 to 0 mm) and numbness (from 100 to 20 mm). MRI showed no local relapses at the surgical incision. The lower extremity pain relief was maintained and was satisfactory during the postoperative course. Radiation therapy was performed to treat the brain metastasis after the surgery. The patient died 9 months postoperatively of Trousseau syndrome and brain metastasis due to breast cancer.

## Discussion and conclusions

Intradural metastases are relatively rare compared with metastases to the vertebral column. The few reports of intradural metastases indicate that the most common primary site is the lung (40–85%), followed by breast cancer and renal cell cancer [2, 6, 8–10]. In most cases, the diagnosis of intradural metastasis is relatively straightforward because the primary site has already been treated with surgery. By contrast, in our patient, cancer in the right breast was cured because appropriate surgery had been performed 30 years ago and there had been no metastases. Therefore, we first diagnosed the mass as a solitary intradural extramedullary cauda equina tumor suspected of being a schwannoma. However, after checking the patient’s general status, we considered the possibility of a metastatic cauda equina tumor from cancer in the left breast. Our patient’s case is considered quite rare because the initial symptoms were low back and lower extremity pain presenting as cauda equina syndrome. To our knowledge, there are only two reports in the literature of spinal metastasis of occult lung cancer causing cauda equina syndrome [2, 11]. According to one of the reports, as in our patient’s case, MRI of the intradural metastasis in the cauda equina appeared similar to that of intradural nerve sheath tumor, and a diagnosis by MRI alone was difficult because the primary lesion was unknown [2]. In addition, in our case, myelography was performed before surgery. A previous report indicated the utility of myelography in lumbar canal stenosis [12]. Furthermore, when planning surgery for spinal cord tumors such as intradural schwannoma, myelography is useful to confirm respiratory fluctuation of the tumor. If a respiratory fluctuation of the tumor is observed, the wide range of laminectomy is needed. In our patient’s case, respiratory fluctuation of the tumor was not observed, and it was difficult to differentiate schwannoma from cauda equina metastasis. Nevertheless, our patient’s case indicates that we should consider the possibility of metastasis in planning surgery for extirpation of a tumor in the cauda equina.

Four pathways for metastatic tumor spread to the spine are hypothesized as follows: hematogenous dissemination (via an artery), through the paravertebral plexus of veins (Batson’s plexus), direct invasion of the bone, and dissemination through cerebrospinal fluid [2, 9, 13, 14]. Generally, metastatic tumors are entrapped by the lung in the hematogenous dissemination pathway. Indeed, multiple metastases to the lung were observed in our patient. In addition, only one small lesion of brain metastasis was found in our patient, which suggested that the pathway through cerebrospinal fluid was not primal. Hence, we speculate that the pathway for metastasis to the cauda equina is through Batson’s plexus and that that resulted in the metastasis to the brain.

Intradural metastases are a form of terminal stage cancer. The survival rate is in the range of 6–9.4 months despite extirpation surgery [15]. Our patient died 9 months after surgery, which is consistent with other reports. However, the patient was quite satisfied with the surgery because her low back and lower extremity pain was relieved substantially. Surgical treatment may be a favorable option to improve the quality of life in such cases, even if the patient’s prognosis is not good [16].

In conclusion, it is difficult to clarify whether tumors in the cauda equina are benign or malignant on the basis of MRI findings alone. Clinicians should consider the possibility of metastasis when planning surgery to extirpate tumors from the intradural cauda equina. Although the prognosis for such metastatic tumors in the cauda equina is not good, surgical treatment should be considered if the patient has severe pain that prevents an acceptable quality of life.

## Data Availability

All the data supporting our findings is contained within the report.

## Abbreviations

CT: Computed tomography
MRI: Magnetic resonance imaging
VAS: Visual analogue scale
